# RNA editing in the forefront of epitranscriptomics and human health

**DOI:** 10.1186/s12967-019-2071-4

**Published:** 2019-09-23

**Authors:** Theodoulakis Christofi, Apostolos Zaravinos

**Affiliations:** 1 Department of Life Sciences, School of Sciences, European University Cyprus, 2404 Nicosia, Cyprus; 2 Centre for Risk and Decision Sciences (CERIDES), 2404 Nicosia, Cyprus

**Keywords:** RNA editing, APOBEC, ADAR, AID, Cytidine/adenosine deaminases, Epitranscriptomics, Cancer, Post-transcriptional modifications

## Abstract

Post-transcriptional modifications have been recently expanded with the addition of RNA editing, which is predominantly mediated by adenosine and cytidine deaminases acting on DNA and RNA. Here, we review the full spectrum of physiological processes in which these modifiers are implicated, among different organisms. Adenosine to inosine (A-to-I) editors, members of the ADAR and ADAT protein families are important regulators of alternative splicing and transcriptional control. On the other hand, cytidine to uridine (C-to-U) editors, members of the AID/APOBEC family, are heavily implicated in innate and adaptive immunity with important roles in antibody diversification and antiviral response. Physiologically, these enzymes are present in the nucleus and/or the cytoplasm, where they modify various RNA molecules, including miRNAs, tRNAs apart from mRNAs, whereas DNA editing is also possible by some of them. The expansion of next generation sequencing technologies provided a wealth of data regarding such modifications. RNA editing has been implicated in various disorders including cancer, and neurological diseases of the brain or the central nervous system. It is also related to cancer heterogeneity and the onset of carcinogenesis. Response to treatment can also be affected by the RNA editing status where drug efficacy is significantly compromised. Studying RNA editing events can pave the way to the identification of new disease biomarkers, and provide a more personalised therapy to various diseases.

## Introduction

### The discovery of RNA editing and the field of Epitranscriptomics

RNA modifications refer to alterations in the chemical structure of RNA molecules occurring after DNA transcription and synthesis by the RNA polymerase enzyme. They were first described in 1968 with the discovery of RNA methylation in Hela cells [1]. Since then, modifications have been observed across many RNA types (miRNA, mRNA, rRNA, etc.) and detected in all domains of life, including archaea, prokaryotes and eukaryotes. So far, 112 nucleotide modifications have been observed, with the potential to affect the function and stability of the RNA molecule [2].

**Fig. 1:**
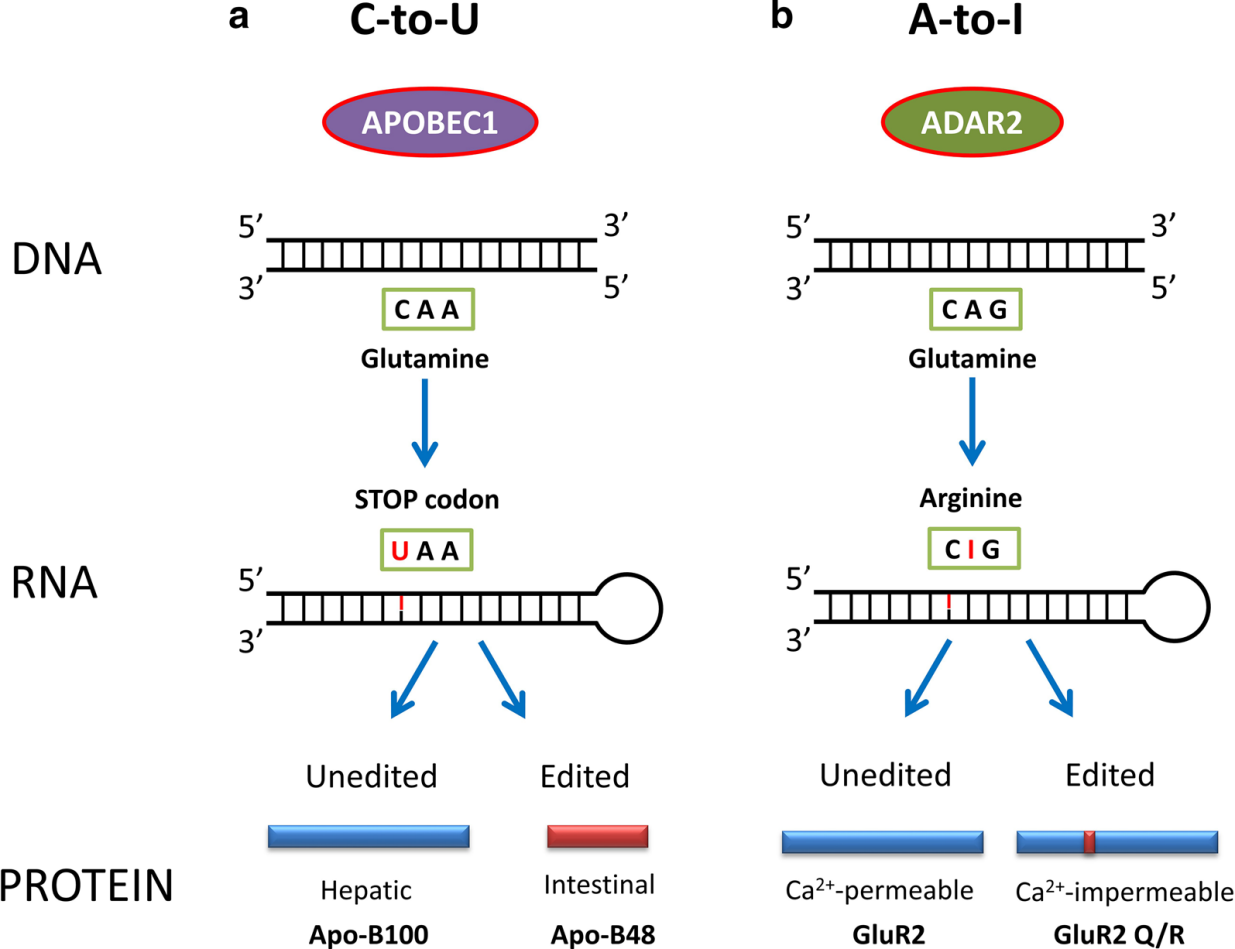
Cytidine and adenosine deaminases are critical RNA editors that play important functions in physiological events. **a** The vital role of APOBEC1 editing can be observed in the production of apolipoprotein B in the gut. The C-to-U editing at residue 2153 of hepatic Apo-B100 transforms the glutamate to a stop codon and produces a truncated protein Apo-B48 in intestinal cells [4]. **b** In neurons, mRNA editing of the glutamate receptor 2 (GluR2) at position 607 by ADAR2 results in an adenosine to inosine chance. This transforms the C**A**G codon for glutamine (Q) to C**I**G for arginine (R) as (C**G**G), since ribosomes read inosine (I) as guanosine (G). This neutralizes the diffusion of divalent cations and makes the receptor impermeable to calcium [112]

A unique type of RNA modification in trypanosome mitochondrial mRNA was discovered ~ 30 years ago [3]. The highly conserved mitochondrial cytochrome c oxidase subunit II (COX-2) gene mRNA was found to have four extra uridine (U) nucleotides, which could restore the reading frameshift to a functioning gene transcript. This post-transcriptional modification which edits the RNA transcript sequence, differentiating it from its corresponding DNA sequence, was named RNA editing. A year later, evidence of tissue-specific RNA editing was discovered in mammals [4]. The production of apolipoprotein-B48 in the intestine was observed to occur after a post-transcriptional cytidine to uridine (C-to-U) mRNA change in the gene’s transcript, which is responsible for the production of the hepatic apolipoprotein-B100. This change creates a translational stop codon and the functionally truncated intestinal protein (Fig. 1a).

Early characterisation of RNA was time consuming and required substantial sample for sequencing because of its fragile state. In the beginning, researchers combined the knowledge of RNase enzymes to cut the RNA at specific sites and fragments, leading to the first complete tRNA sequence of *Saccharomyces cerevisiae* alanine tRNA [5, 6]. In 1977, alternative splicing was observed in adenoviruses demonstrating the capability of post-transcriptional modifications [7, 8]. Continued technological innovation led to the emergence of next-generation sequencing (NGS) technologies and made possible the high-throughput sequencing and identification of numerous RNA editing sites [9]. Furthermore, the programming of powerful computational methodologies enabled the study and prediction of RNA editing to become much more feasible [10].

The field of post-transcriptional RNA modifications is expanding beyond the common view of adjusting the structures and functions of mature RNA. Named RNA Epigenetics, or Epitranscriptomics, is an increasing group of RNA modifications classified in 4 groups: (1) the isomerization of uridine to pseudouridine, (2) alterations in the bases, including methylation and deamination, (3) methylation of the ribose 2′-hydroxyl (-OH) group and (4) complex and multiple modifications or hypermodifications [11]. Epitranscriptomics is progressively associated with many biological functions, from brain development and neuronal regulation to antibody diversification and immune defence [12, 13].

### Conservation among species and the “Constructive Neutral Evolution” proposal

The identification of an increasing number of RNA edited sites in many organisms signifies the importance of this phenomenon in the evolution of species. The presence of RNA editing has been well observed in plants and animals [14, 15]. Phylogenetic analysis of the deaminase enzymes responsible for RNA editing, suggests that adenosine and cytidine deaminases emerged early in the metazoan radiation [15–17]. It is believed that they arose from an early transfer of an ancestral deaminase from bacteria toxin systems [18, 19]. Supporting the above hypothesis, recent evidence shows that RNA editing contributes in central processes in bacteria, thus, regulating evolutionarily conserved toxin-antitoxin systems [19]. From fruit flies to humans, RNA editing affects multiple cellular processes and is highly conserved [15, 20, 21].

The central dogma of molecular biology states that information is transferred from DNA to RNA to protein. Why RNA editing has emerged is a question that puzzled scientists, and is still under debate [22]. Simple answers are to repair genomic mutations or to provide another level of protein abundance. A three step model has been proposed for the development of RNA editing, accounting for the emergence of its activity, the mutation of editable nucleotide positions and fixation by drift, leading to its maintenance by natural selection [23]. This model has been expanded further, to describe how RNA editing could have evolved in the absence of positive selection [24]. Neutral theory suggests that neutral mutations and genetic drift account for the evolutionary changes at the molecular level [25]. Constructive neutral evolution (CNE) proposes that RNA editing systems emerged in existing proteins with metabolic activity where they remain active due to their ability to fix deleterious mutations in the RNA level. This can lead to an accumulation of gene mutations, since functional constrains are suppressed. However, RNA editing can be lost if these gene mutations are reversed; or if they accumulate, it becomes essential for the flow of genetic information [22, 26].

## Deaminases acting as editors

### Adenosine to inosine (A-to-I) editors

The unwinding of double stranded RNA (dsRNA) in oocytes of *Xenopus laevis* was the first evidence of adenosine to inosine (A-to-I) RNA editing [27]. The number of adenosine deaminases acting on RNA (ADARs) has increased, since then [28]. A-to-I conversion is the most common type of RNA editing [29]. In mammals, three ADAR genes (ADAR1-3) produce four isoforms, ADAR1p150, ADAR1p110, ADAR2 and ADAR3 [30, 31]. ADAR1 and ADAR2 arose from gene duplication around 700 million years ago in the early metazoan evolution [32]. ADAR3 most likely arose within the vertebrate linage from ADAR2 gene duplication [32]. The genome of *Caenorhabditis elegans* harbours two ADAR genes, adr-1 and adr-2 [33], while the genome of *Drosophila melanogaster* has only one, dADAR, that is under strict developmental control [34]. Two more ADAR-like genes (renamed to ADAD) are found in vertebrates; ADAD1 (or testes nuclear RNA-binding protein, TENR) which is required for spermatogenesis, and ADAD2, which is expressed in the brain [35, 36]. TENR-like genes have also been observed in the genomes of *Danio rerio and Takifugu rubripes* [32].

Adenosine deaminases that act on tRNAs (ADATs) form another class of A-to-I enzymes targeting tRNA molecules, and are believed to be evolutionary ancestors of ADARs [37]. In bacteria, TadA was the first prokaryotic RNA editing enzyme characterised in *Escherichia coli* [38]. ADATs have also been observed beyond metazoan, and are found in many prokaryotic and eukaryotic organisms [16]. In most eukaryotes, including humans, three ADAT enzymes (ADAT1-3) have been identified [39].

### Cytidine to uridine (C-to-U) editors

Cytidine deaminases within the activation induced cytidine deaminase/apolipoprotein B editing complex (AID/APOBEC) family are responsible for the C-to-U mRNA editing, but also for DNA editing of deoxycytidines to deoxyuridines (dC-to-dU) [40, 41]. It was previously thought that the AID/APOBEC family of deaminases (AADs) was restricted to vertebrates. Nevertheless, further evidence showed that APOBEC-like proteins are also present across diverse metazoan dictyostelid, and algal lineages [17, 42].

In humans, 11 AADs are expressed, including AICDA (AID), APOBEC1, APOBEC2, APOBEC3A, APOBEC3B, APOBEC3C, APOBEC3D, APOBEC3F, APOBEC3G, APOBEC3H and APOBEC4. Phylogenetic analysis predicts that AID and APOBEC2 emerged in jawless fish (agnatha) ~ 500 million years ago, whereas APOBEC1 emerged in birds and reptiles ~ 300 million years ago. Gene duplication and divergence gave rise to the APOBEC3 subgroup in mammals ~ 100 million years ago, while APOBEC4 seems to have appeared ~ 20 million years ago [43]. Evolutionary analysis however, suggests that AADs emerged from bacterial lateral transfer; and are divided into secreted deaminases (SNADs) and classical AADs, which diversified and evolved rapidly with a widespread distribution across the tree of life [42, 44]. A unique example of cytidine deaminase acting on tRNA base C8 (CDAT8) enzyme was found in archaea, in *Methanopyrus kandleri* [45].

### Alternative U-to-C and G-to-A editing

RNA editing can also occur in the form of U-to-C and G-to-A, called “alternative mRNA editing”. U-to-C was initially identified in the mRNA of Wilm’s tumor 1 (WT1) human transcript [46]. G-to-A editing is another alternative editing process, detected in the heterogeneous nuclear ribonucleoprotein K (hnRNP K) protein of colorectal cancer and surrounding tissues [47]. The precise mechanism of these RNA editing types is still under investigation. However, recent evidence suggests that APOBEC3A is implicated in G-to-A editing in WT1 transcripts, opening the door for unravelling these alternative processes [48].

### Cellular localisation and tissue specificity of RNA editing

From the very beginning of the discovery of RNA editing, it was evident that this modification process can be tissue-specific. The hepatic apolipoprotein-B100 was found to be truncated in enterocytes through a C-to-U change and production of the intestinal apolipoprotein-B48 [4]. In cockroaches, U-to-C and A-to-I editing events of the sodium channel gene (*BgNa*_*v*_) were observed in a tissue/cell-specific (ovary, gut, leg and nerve cord) and development-specific manner, thus, generating functional variants of sodium channels [49]. In plants, > 100 C-to-U edits were found in grape mitochondria, whereas 28% of them are significantly tissue-specific [50].

The regulation of RNA editing and tissue specificity can be closely observed during development [51]. For instance, ADAR2 deficient mice exhibit reduced RNA editing activity and are prone to seizures and early mortality [52]. In addition, Adar^5G1^ null mutant flies lack editing events in hundreds of central nervous system (CNS) transcripts and have defective locomotion, neurodegeneration and reduced survival [53]. In *Drosophila*, mice and human studies, there were significantly more A-to-I edits found in the brain compared to other tissues [54–56]. In the global scale, ADAR1 is the primary editor of repetitive sites (i.e., Alu repeats), ADAR2 is the primary editor of non-repetitive coding sites, while ADAR3 mainly inhibits editing [57]. On the other hand, ADATs seem to be expressed ubiquitously in human tissues [58].

In the cell, pre-mRNA editing is often constrained in the nucleus, hence the localisation of most ADAR enzymes. ADAR1 and ADAR2 are in steady motion all through the nucleolus and are recruited at regions of editing substrate accumulation like in the nucleoplasm [59]. ADAR1p150 is the most common ADAR1 isoform to be found outside the nucleus, trafficking between nucleus and cytoplasm [60]. It can bind to Exportin-1 and translocates in the cytoplasm where it edits (A-to-I) the 3′UTR of dsRNAs [61]. The ADAR1p110 isoform can also shuttle between the nucleus and cytoplasm, but it is predominantly nuclear and constitutively expressed [62]. ADAR2 is considered to be nuclear, since it is rapidly degraded by E3 ubiquitin ligase WWP2 in the cytoplasm, while ADAR3 appears to transiently translocate from the cytoplasm to the nucleus upon neuronal activation [63, 64].

The flux of AADs from the nucleus to the cytoplasm and reverse, is characterised by substantial tissue specificity [41]. AID is expressed in pluripotent tissues like embryonic germ and stem cells and oocytes [65]. APOBEC1 is primarily observed in the intestine of most mammals [66]. APOBEC2 is expressed in the skeletal muscle and cardiac tissue where it is essential for the development of muscles [67]. The subgroup of APOBEC3A-G proteins are heavily implicated in antiviral innate immunity; hence, they are found in immune cell populations [68]. Each family member has different subcellular localisations; namely APOBEC3A, APOBEC3C and APOBEC3H can be either nuclear or cytoplasmic, APOBEC3D, APOBEC3F and APOBEC3G are cytoplasmic, while APOBEC3B is mainly nuclear [69]. APOBEC4 is probably expressed in testes [70].

### Cytidine/adenosine deaminase structural features and RNA targets

**Fig. 2:**
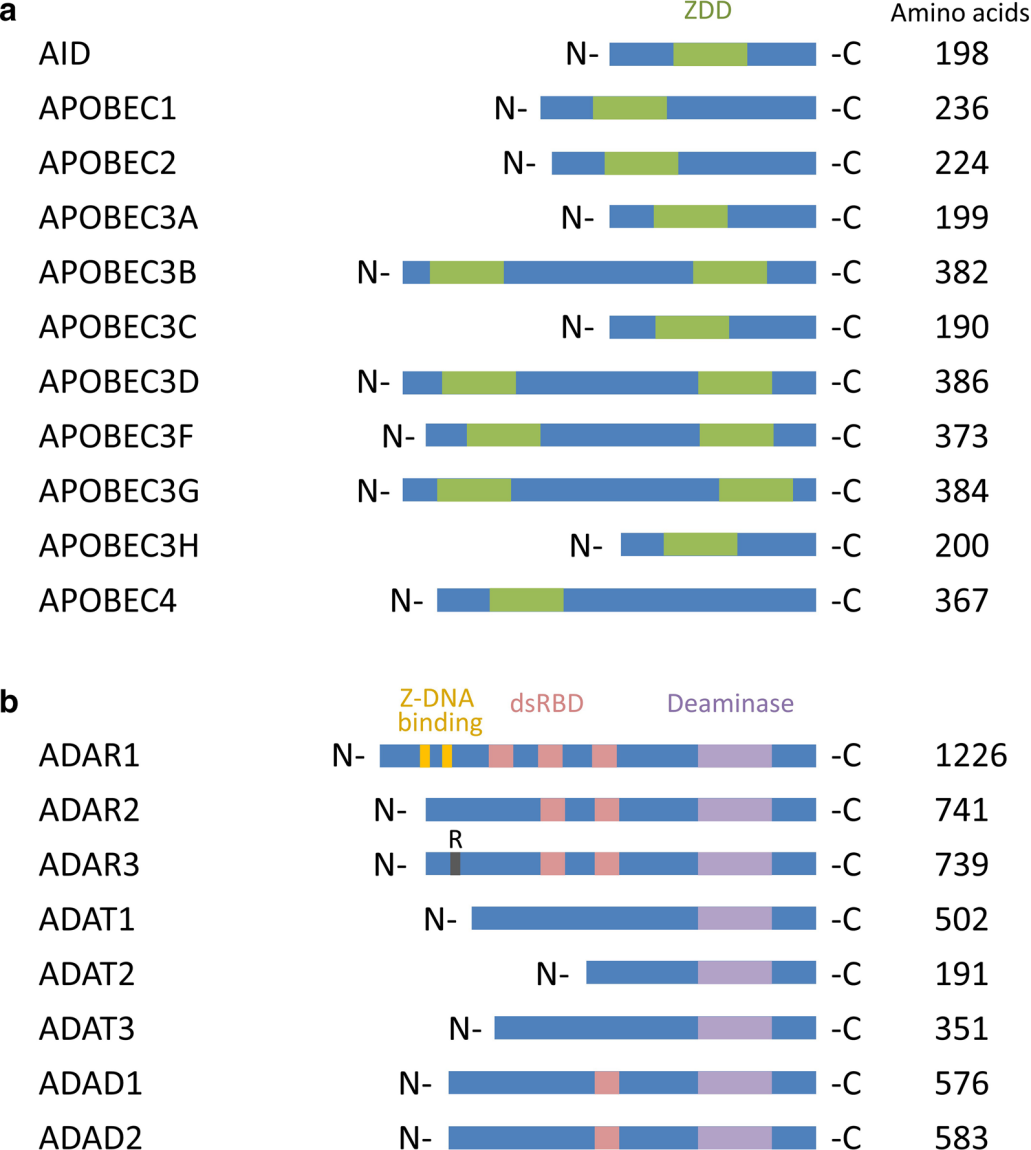
Human cytidine and adenosine deaminase family members. **a** The cytidine deaminase AID/APOBECs family is shown. Activation-induced cytidine deaminase (AID or AICDA) and all apolipoprotein B mRNA editing enzyme-catalytic polypeptide-like (APOBECs) have one catalytically active cytidine or deoxycytidine deaminase domain (ZDD). APOBEC3 diversifies in 7 submembers (APOBEC3A, APOBEC3B, APOBEC3C, APOBEC3D, APOBEC3F, APOBEC3G, APOBEC3H) whereas some have dual deaminase domain structures but the one in the N terminus is catalytically inactive. **b** The adenosine deaminase ADARs, ADATs and ADADs families are shown. Three members of the adenosine deaminase acting on RNAs (ADAR1, ADAR2, ADAR3). Two isoforms are known for ADAR1, ADAR1-p150 and ADAR1-p110 and harbour Z-DNA-binding domains. ADAR3 has a unique arginine-rich R domain. Three members of the adenosine deaminase acting on tRNAs (ADAT1, ADAT2 ADAT3). Up to three repeats of the dsRNA binding domain (dsRBD) and a catalytic deaminase domain are present in adenosine deaminases. Two adenosine deaminase domain-containing proteins (ADAD1, ADAD2) are also known as TENR and TENRL respectively. Amino acid length and motifs retrieved from UniProt database [198]. Length is not drawn to scale

All AADs have a distinct zinc-dependent cytidine (C) or deoxycytidine (dC) deaminase domain (ZDD) (Fig. 2a) [41]. Most of them (AID, APOBEC1, APOBEC2, APOBEC3A, APOBEC3C, APOBEC3H and APOBEC4) have one ZDD while the others (APOBEC3B, APOBEC3D, APOBEC3F and APOBEC3G) have two ZDD domains, in tandem. APOBEC proteins with two ZDDs have a catalytically active C-terminal domain, and an inactive N-terminal domain [71]. APOBEC1 requires a trans-acting element, RNA binding protein cofactor (A1CF) or RNA-binding protein RBM47, and a cis-acting motif composed of 11 nucleotides, termed the mooring sequence [72–74].

In humans, three ADAR enzymes are expressed (Fig. 2b). ADAR1 has two splice variants, ADAR1p110 and ADAR1p150. Three dsRNA binding domains (dsRBD) are found in ADAR1, and two in ADAR2 and ADAR3, respectively [75]. The C-terminal regions are catalytically active but a homodimerization is needed for A-to-I activity [76]. ADAR1 also contains two Z-DNA binding domains, which are required for its localisation to stress granules [77]. An arginine-rich single-stranded RNA (ssRNA) binding domain (R domain) is uniquely present in the N-terminal region of ADAR3 [78].

RNA editing occurs primarily within noncoding regions, and only a small percentage takes place in coding regions resulting in amino acid change. In humans, A-to-I editing mostly occurs in introns and untranslated regions (UTRs) of protein coding genes [79]. A-to-I editing sites seem to happen more frequently in 3′ UTRs than in 5′-UTRs [80, 81]. Alu repeats, a repetitive short interspersed element (SINE), is the most favorable target of RNA editing. Up to 700,000 Alu elements are present in humans and can harbor at least 100 million A-to-I editing sites [82].

MicroRNAs (miRNAs) are also processed by ADARs (ADAR1 and ADAR2) for A-to-I editing in order to control miRNA biogenesis [83, 84]. In addition, evidence of RNA editing competition with RNA interference (RNAi) dsRNA substrates, suggests that ADARs act as modulators of the RNAi machinery [85]. Furthermore, tRNA can be edited by ADATs, as mentioned above. In humans, A-to-I edits at positions 34 and 37 of tRNA^Ala^ have been reported [86]. Even though widespread in plants, rRNA editing has not yet been observed in humans [87].

APOBEC1 has a preference for AU-rich sequences within mRNA 3′ UTRs [88]. APOBEC3 proteins are significantly active against endogenous retroelements and retroviruses, where they can target SINE RNA but also long interspersed nuclear elements (LINEs) [89, 90]. APOBEC2 and APOBEC4 are still under investigation since little evidence have emerged for their activities [70, 91, 92].

### Target detection by next-generation sequencing (NGS) and computational processing

Classical molecular biology and sequencing techniques have contributed significantly in the discovery and early detection of RNA editing. The emergence NGS technologies in combination with accurate bioinformatic pipelines made possible the detection of thousands of new RNA editing sites [81, 93]. Matched DNA and RNA sequencing in single samples identifies RNA–DNA differences that can provide a plethora of possible edits [79, 94].

High-throughput sequencing methods like RNA-seq have now become readily available in the scientific community, leading to a wealth of accumulating transcriptomic data of various tissues and cell populations [95]. This extensive repertoire of information and the above techniques demand extensive computational tools. Besides software packages that identify RNA editing sites from matched sequencing samples, increasing programming is given to prediction models. Candidate RNA editing sites can be predicted by software packages like GIREMI (Genome-independent Identification of RNA Editing by Mutual Information, https://github.com/zhqingit/giremi), RNAEditor (http://rnaeditor.uni-frankfurt.de/) and DeepRed (https://github.com/wenjiegroup/DeepRed) from RNA-seq data in the absence of relevant genomic data [10, 80, 96].

### RNA editing databases

Furthermore, freely available databases are now available, where one can explore RNA editing collections, in humans and other model organisms. The RNA Editing ATLAS (https://omictools.com/the-rna-editing-atlas-tool) is the first human inosinome atlas, comprising > 3.0 million A-to-I events identified in six tissues from three healthy individuals. Matched directional total-RNA-Seq and whole genome sequence datasets were generated and analysed within a dedicated computational framework, also capable of detecting hyper-edited reads. Inosinome profiles within the RNA Editing ATLAS are tissue specific and edited gene sets, consistently show enrichment of genes involved in neurological disorders and cancer. The RNA Editing ATLAS reports that overall, the frequency of editing varies, but is strongly correlated with the expression levels of ADAR [97].

Moreover, the RADAR database (http://rnaedit.com/) presents a comprehensive collection of A-to-I editing sites in human, mouse, and fly transcripts [98].

dbRES is another RNA editing database that contains only experimentally validated data that were manually collected from literature reporting related experiment results or from GeneBank [99].

REDIportal (http://srv00.recas.ba.infn.it/atlas/) is another freely available database collecting > 4.5 millions of A-to-I editing events in 55 body sites of 150 healthy individuals from the GTEx project. In REDIportal, RNA editing sites can be searched by genomic region, gene name and other relevant features as the tissue of origin. Recently, REDIportal started collecting A-to-I events in non-human organisms [100].

REDIdb in another RNA editing database, where one can assess general information, editing features and the nucleotide genomic sequence and the corresponding transcript of the entry, annotated as cDNA [101].

Furthermore, LNCediting (http://bioinfo.life.hust.edu.cn/LNCediting/) provides a comprehensive resource for the functional prediction of RNA editing in long noncoding RNAs (lncRNAs) in *Homo sapiens*, *Macaca mulatta*, *Mus musculus* and *Drosophila melanogaster* [102].

## Role in health

### Modulators of alternative splicing and transcriptional control

**Fig. 3:**
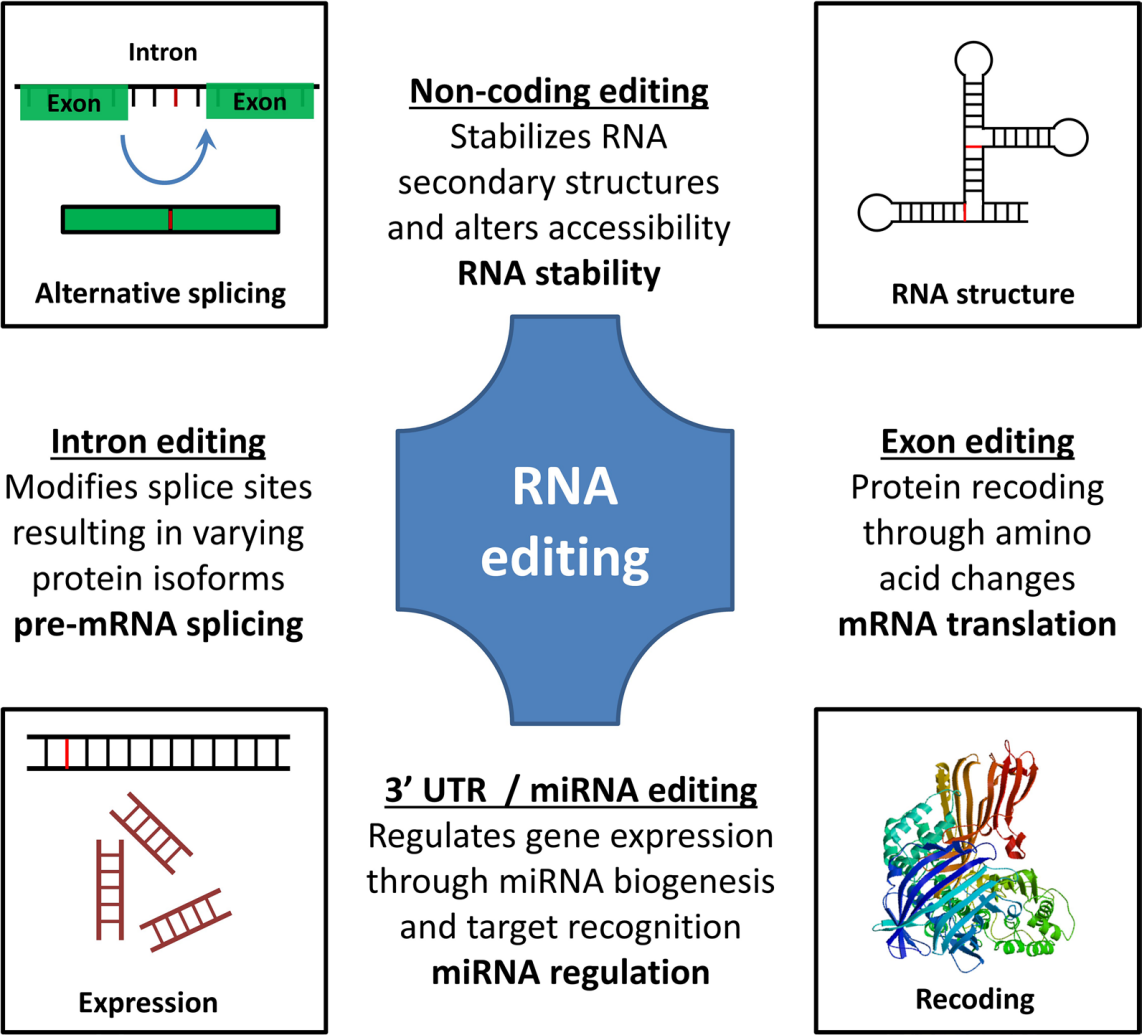
Functional roles of RNA editing. Adenosine and cytidine deaminases target RNAs molecules and modify their sequence affecting multiple processes. A-to-I or C-to-U modifications in RNA transcripts are reflected in the folding of the RNA structure influencing its structural stability and binding accessibility for further processing [199]. mRNA abundance and gene expression is regulated by miRNA or miRNA target editing, influencing gene silencing by RNA degradation [84, 156]. Protein diversity is another outcome of RNA editing since it can create or abolish splicing sites regulating alternative splicing [54]. Moreover, editing in coding regions can recode amino acids and create an alternative protein with distinct functionalities [4]. Recoding protein example was built with SWISS-MODEL workspace [200]

RNA editing can result in a number of functional effects (Fig. 3). A-to-I editing can produce or delete splice sites, regulating the production of different protein isoforms with varying traits [103–105]. For instance, in neurons RNA editing regulates synaptic transmission through editing and splicing of glutamate receptor pre-mRNA [103, 104]. Sequencing the RNA of different subcellular fraction showed that most (> 95%) of the A-to-I edits happen during mRNA maturation with ~ 500 editing sites in the 3′ acceptor sequences [106]. Modifications in these splice sites can easily result in alternative splicing of the associated exons. Interestingly, ADAR itself is regulated by RNA editing in order to produce the *Drosophila* dADAR variants [34]. In rats, ADAR2 edits twice its own pre-mRNA to produce four isoforms in order to control its own expression [105]. Moreover, ADAR1 and ADAR2 are related to spliceosomal proteins [107]. A nice example of RNA editing-mediated splice variant is the nuclear prelamin A recognition factor (NARF) exon 8 evolution in primates [108].

### RNA editing regulates neuronal dynamics

Studies in animal models (*Drosophila* and mice) implicated ADARs and A-to-I editing post-transcriptionally regulates circadian rhythm and sleep [109, 110]. ADAR1 deficiency in *Drosophila* led to constitutive release of neurotransmitter in glutamatergic neurons promoting sleep [111]. RNA editing association with neuronal activity is supported by the fact that ADAR2 and ADAR3 are generally highly expressed in the brain and CNS [64, 78]. During development and normal function, RNA editing acts as a regulator of neurotransmission and signal transduction by editing AMPA and kainate glutamate receptor subunits at the Q/R site making them impermeable to Ca^2+^, whilst regulating Ca^2+^ influx; a prerequisite for normal neuronal function (Fig. 1b) [112]. Additional properties (like targeting miRNA and circular RNA) have been attributed to RNA editing strengthening its role as a powerful and dynamic regulator of neuronal function, brain development and protection [113–115].

### Fundamental players in innate and adaptive immunity

B lymphocytes produce a repertoire of antibodies in order to protect the organism from foreign and invading pathogens. This process is facilitated by genetic mechanisms such as gene rearrangement and conversion, somatic hypermutation (SHM) and class switch recombination (CSR). AID plays an important role in antibody diversity through both SHM and CSR [116]. Two models have been proposed for AID activity in immunoglobulins, DNA or RNA editing [117]. Most evidence however, support the notion that AID targets DNA hotspots; like the WRC motif of the variable region (where W = A or T and R = A or G) or the DGYW/WRCH motif (where G:C is the mutable position; D = A/G/T, H = T/C/A) of switch (S) region sequence causing U:G mismatches [118, 119]. These, either become point mutations and SHM or trigger double strand breaks (DSB) and CSR, respectively [119, 120].

APOBECs are heavily implicated in viral immune defense. APOBEC3G is the first family member that was identified as an antiviral factor and the most well studied [121, 122]. In the human immunodeficiency virus (HIV-1), APOBEC3G deaminates C-to-U in the reverse transcribed viral cDNA minus strand, causing G-to-A hypermutation on the plus strand that can lead to cDNA instability and inhibition of viral replication [123]. APOBEC3D, APOBEC3F and APOBEC3G in humans, and and APOBEC3H in the rhesus macaque are also shown to inhibit HIV-1 [124]. However, the HIV-1 virion infectivity factor (Vif) can ubiquinate APOBEC3 proteins targeting them for proteasomal degradation [123, 124].

Different levels of antiretroviral activity among APOBECs have also been demonstrated against Simian immunodeficiency virus (SIV), adeno-associated virus type 2 (AAV-2), murine leukemia virus (MLV), equine infectious anemia virus (EIAV), and foamy viruses (FVs) [89, 125–128]. Furthermore, hepatitis B virus (HBV) replication can be hampered by APOBEC3G in a deamination-dependent and -independent manner [129, 130].

Apart from viral C-to-U DNA editing, APOBEC3-mediated C-to-U RNA editing is also observed in HIV genomic RNA [131]. APOBECs also target RNA viruses such as members of the paramyxoviruses, measles and mumps, but also respiratory syncytial viruses (RSV) by C-to-U hypermutations [132].

ADARs (especially ADAR1 isoforms) target RNA viruses like measle virus, infuenza A virus, Rift Valley fever virus (RVFV), lymphocytic choriomeningitis virus (LCMV) and hepatitis C virus (HCV) by A-to-I hypermutation [133–137]. However, they also exhibit a significant degree of proviral effects (reviewed in [138]).

AID/APOBEC proteins have the capacity to restrict LTR and non-LTR retrotransposons [90, 139]. Retrotransposons are genetic elements able to transport themselves and multiply in the genome. LTR retrotransposons are mainly inhibited by APOBEC3 proteins through C-to-U DNA hyperediting [140]. Long interspersed nuclear element 1 (LINE-1) is the only functional family of transposable elements in humans [141]. Accumulating evidence supports that LINE-1 retrotransposon inhibition by AID/APOBECs and ADARs is editing-independent (reviewed in [142, 143]). Non-autonomous SINE-1 is another member of the non-LTR retrotransposons. The SINE Alu repeats exhibit strong A-to-I editability by ADARs and account for the majority of all editing sites [82, 144].

## Role in disease

### Aberrant involvement in human diseases

The contribution of RNA editing in the brain and CNS is not always beneficial. Evidence have emerged implicating RNA editing in the pathogenesis of neurodegenerative diseases like amyotrophic lateral sclerosis (ALS), Parkinson and Alzheimer diseases [145]. In ALS, mutation of the optineurin (OPTN) gene was found to be triggered by recombination between Alu elements, a favourable target of A-to-I editing [146]. In addition, down-regulation of ADAR2 is detrimental towards the physiological editing of glutamate receptor Q/R site and calcium regulation, leading to neuronal hyper-excitability and autophagy, which can contribute to the death of motor neurons in ALS [147, 148]. Reduction of glutamate receptor editing was also observed in Alzheimer disease [149]. A number of studies investigated editing patterns of the serotonin 2C receptor in patients with various psychological disorders like schizophrenia, depression, bipolar disorder, drug addiction and even in individuals who committed suicide (reviewed in [150]). Despite significant associations, results are conflicting and inconclusive requiring further investigation. ADAR mutations and altered editing have also been linked to autoimmune diseases like Aicardi-Goutières syndrome and systemic lupus erythematosus, respectively [151, 152].

### RNA editing in cancer

Cancer pathogenesis is primarily attributed to genetic mutations in proto-oncogenes and tumour suppressor genes, which transform a normal cell into a malignant cancer cell. The rapid progress of NGS technologies and the readily available transcriptomic data have revealed a significant contribution of RNA editing in the pathogenesis and progression of cancer (Table 1) [153, 154]. Transcriptome analysis of various tumours showed deferential RNA editing levels depending on the cancer type. Decreased A-to-I editing patterns have been identified in brain, kidney, lung, prostate and testis tumours, with significant global hypo-editing of Alu elements [155]. On the other hand, recent studies on multiple cancer tissues found elevated editing levels in intergenic, intronic and 3′ UTR regions of most cancer types especially in thyroid, head and neck, breast and lung cancer tissues compared to their matched controls, which in most cases is associated with worst patient survival [153, 154]. These data clearly indicate that the editing level, high or low, can have different roles in the pathogenesis of cancer and different clinical outcomes in the progression of the disease.

**Table 1:** RNA editing events in cancer

Editors	Cancer type	Target	Effect	Organism	Study
A-to-I	Breast	GABRA3, Akt*	Promotes migration, invasion and metastasis	Humans/cell lines/mice	[164]
A-to-I	Breast	COPA	Increases proliferation, invasion and migration	Humans/cell lines	[165]
ADAR1, ADAR2	Gastric	PODXL	Drives tumorigenesis and progression	Humans/cell lines/mice	[166]
ADAR2	Glioblastoma	miR-222/221, miR-21	Controls cell proliferation and migration	Humans/cell lines/mice	[157]
ADAR2	Glioblastoma	miR-376a, RAP2A*	Inhibits invasion and migration	Mice/cell lines	[158]
A-to-I	Colorectal	RHOQ	Promotes invasion	Humans/cell lines	[168]
ADAR2	Esophageal	IGFBP7	Inhibits apoptosis	Humans/cell lines/mice	[169]
ADAR2	Esophageal	SLC22A3, ACTN4*	Promotes invasion and metastasis	Humans/cell lines/mice	[170]
ADAR2	Glioblastoma	CDC14B, Skp2/p21/p27*	Inhibits tumour growth	Humans/mice	[167]
ADAR1	Cervical	BLCAP, STAT3*	Drives tumorigenesis and progression	Humans/cell lines	[174]
ADAR1	Liver	AZIN1	Tumorigenesis	Humans/cell lines/mice	[171]
ADAR1	Esophageal	FLNB, AZIN1	Aggressive tumour behaviour	Humans/cell lines	[172]
ADAR1	Colorectal	AZIN1	Oncogenic potential and cancer stemness	Humans/cell lines/mice	[173]
ADAR1	Lung	miR-381, NEIL1	Cancer stemness and chemoresistance	Cell lines/mice	[160]
ADAR1	Melanoma	miR-455-5p, CPEB1*	Inhibits cancer growth and metastasis	Mice/cell lines	[159]
ADAR2	Colorectal	miR-200	Promotes liver metastasis	Humans/cell lines/mice	[163]
AID	Lymphoma, Leukemia	CSR, SHM, c-myc*, notch1*, Ebf1*, Pax5*	Mutagenic potential and tumorigenesis	Mice/cell lines	[175–177]
AID	Gastric	CDKN2a, CDKN2b	Tumorigenesis	Humans/Mice	[178]
APOBEC1	Esophageal, Leukemia	n/a, BCR-ABL1*	Mutagenic potential	Humans/cell lines	[179]
APOBEC3B	Breast	KATAEGIS, TP53*	Cancer progression and poor outcome	Humans/cell lines	[180, 181]
APOBEC3G	Colorectal	miR-29, MMP2*	Promotes liver metastasis	Humans/cell lines/mice	[182]

Downstream affected genes by the editing event are indicated with an asterisk

As mentioned above, editing sites fall within noncoding regions of the genome, and this is also true for cancer genomes. MiRNAs are also important editing targets in cancer [156]. In glioblastoma, the anti-tumour function of ADAR2 was revealed, as it can regulate a large number of miRNAs, including miR-21 and miR-222/-221 precursors and reduce the expression of their mature oncogenic miRNAs [157]. Edited miR-376a was also found to exhibit anti-tumour effects in glioblastoma by targeting the RAP2A oncogene (a member of RAS oncogene family), while its unedited form targets the autocrine motility factor receptor (AMFR) receptor, thus promoting invasiveness [158]. In melanoma, unedited miR-455-5p promotes cancer growth and metastasis by inhibiting the tumour suppressor gene cytoplasmic polyadenylation element binding protein 1 (CPEB1) [159]. Overexpression of ADAR1 in lung cancer has been associated with poor outcome as it enhances editing of miR-381-associated stemness and chemoresistance, in addition to editing NEIL1, a DNA repair gene [160]. Besides unique miRNA editing events in individual cancer types, a recent study investigated 20 different cancers from The Cancer Genome Atlas (TCGA) and identified 19 RNA editing hotspots [161]. Among them, miR-200b, a tumour suppressor, was found to promote invasion and migration when edited by suppressing LIFR, a characteristic metastasis suppressor; while been associated with worst patient survival. Furthermore, it has been shown that editing of 3′ UTR can regulate miRNA binding sites affecting post-transcriptional regulation of tumour suppressor genes and oncogenes like MDM2 which promoted carcinogenesis [162]. Another study identified the secretion levels of miR-200 family members to be regulated by ADAR2 and protein kinase Cζ (PKCζ) axis in promoting liver metastasis in colorectal cancer [163].

Editing in the protein coding region is far less frequent, but it has major consequences in the regulation and function of the affected gene. Sixty recoding events have been identified in a large scale study that are associated with tumours [153]. Other recoding events include the RNA editing of GABRA3 and COPA in breast cancer, which suppresses AKT-mediated metastasis or promotes proliferation, invasion and metastasis, respectively [164, 165]. Gastric cancers display profound misediting of RNA since they exhibit significant genomic gain of ADAR1 and loss of ADAR2 genes acting as oncogenic and tumour suppressive mediators; hence the failure of ADAR2 recoding of PODXL gene allows its tumorigenic potential [166]. Other RNA editing events on coding sequences include the RHOQ gene in colorectal cancer, IGFBP7 and SLC22A3 in esophageal cancer and CDC14B in glioblastoma [167–170]. The most well characterised recoding event is the editing of the coding sequence of AZIN1 mRNA in cancers [162, 171–173]. In liver cancer, an ADAR1 A-to-I editing of AZIN1 transcripts results in a serine to glycine substitution at residue 367. This affects protein conformation and induces a nuclear translocation leading to tumour initiation potential and progression [171]. In colorectal cancer, AZIN1 RNA editing exhibits cancer stemness and augments oncogenic potential; while in esophageal squamous cell carcinoma it is associated with aggressive tumour behaviour [172, 173].

Cytidine deaminase mutations have also been observed in an array of human cancers [183]. APOBEC misregulation in cancer, contributes to genetic instability and affects prognosis depending on the type of cancer [181, 184]. APOBEC3A and APOBEC3B overexpression has been associated with localised C-to-T and/or C-to-G hypermutations termed “kataegis” in a number of cancers, suggesting that these signatures are important in cancer mutagenesis [183, 185, 186]. In breast cancer, APOBEC3B overexpression has been linked to DNA C-to-U editing and TP53 inactivation while it is correlated with poor treatment response and poor outcome for estrogen receptor-positive (ER+) tumours [180, 181, 187]. Furthermore, increased expression of APOBEC3G in colorectal tumours has been found to promote hepatic metastasis through inhibition of miR-29 mediated suppression of matrix metalloproteinase 2 (MMP2) [182]. However, in bladder cancer, APOBEC-low expressing tumours often present mutations in FGFR3 and RAS family of oncogenes; whereas APOBEC-high expressing tumours usually have mutations in DNA damage response genes and chromatin regulatory genes, an enhanced immune response and better overall survival [184]. C-to-U RNA editing by APOBEC1 is also observed in cancers and is especially enriched in lung tumorigenesis and hepatocellular carcinoma [188, 189].

Another important role of RNA editing in cancer is its involvement in the ability of tumours to evade immune responses. APOBEC overexpression and *kataegis* has been associated with programmed cell-death receptor-1 (PD-1) overexpression, an immune checkpoint molecule, leading to immune tolerance and exhaustion [190]. Conversely, RNA editing can elicit immune responses in tumours through increased levels of edited peptides that act as antigens, stimulating T cell responses [191].

In cancers, RNA editing not only changes the sequence of RNAs and their expression but also contributes to proteomic diversity [165, 192]. Combining the TCGA genomic data and the Clinical Proteomic Tumor Analysis Consortium (CPTAC) proteomic data (https://proteomics.cancer.gov/programs/cptac), Peng and colleagues present evidence that the A-to-I RNA editing events in cancer are manifested in protein diversity of cancer cells through changes in amino acid sequences. These intriguing observations suggest that RNA editing is a novel source of cancer protein heterogeneity.

## Future perspectives in diagnosis and treatment

Increasing evidence proclaims that the levels of RNA editing, along with the expression of adenosine and cytidine deaminases and specifically edited genes (especially tumour suppressors and oncogenes), could all be used as important prognostic biomarkers in the pathogenesis and progression of cancer [165, 166, 171]. Deregulated expression patterns of ADARs and APOBECs observed between tumour and normal tissues, as well as within cancer types, revealed a promising scheme of clinical value towards a better understanding of cancer development and its corresponding treatment [154, 180, 183]. Distinctly edited genes like the ones discussed above, play a significant role in tumour pathophysiology [166, 172, 173]. Treatment strategies have also been challenged by nonsynonymous RNA editing events and expression levels, since they display considerable effects on drug sensitivity, as tamoxifen resistance in ER2+ breast cancers [154, 187]. Interestingly these processes also provide new therapeutic targets. ADAR inhibitors is a novel treatment strategy against ADAR-overexpressing tumours, such as in breast and lung, with positive results [193]. In addition, APOBEC inhibitors are still in the early stages of development, due to their recent involvement in cancer, but increasing interest is been directed towards this aspect, as well [194].

Overall, the modulation and application of RNA editing is an area of great potential. Besides traditional deaminase inhibitors to control expression, molecular tools such as antisense oligonucleotides are potent and selective inhibitors of RNA editing on targeted RNAs [195]. Engineered RNA editing-guided activity is a technique particularly useful for hypo-edited-related diseases, such as in prostate and brain cancers, but also in correcting disease-promoting genetic mutations [155, 196, 197].

## Conclusion

In this review, we have discussed the diverse aspects of RNA editing, from its discovery to physiological function and involvement in human diseases. The road ahead still seems bright for RNA editing with new exciting findings from modern transcriptomics and new therapeutic developments in associated disorders.

## Data Availability

Not applicable.

## Abbreviations

COX-2: cytochrome c oxidase subunit II
C-to-U: cytidine to uridine
A-to-I: adenosine to inosine
NGS: next-generation sequencing
CNE: constructive neutral evolution
dsRNA: double stranded RNA
ADAR: adenosine deaminase acting on RNA
ADAD: ADAR-like deaminase
TENR: testes nuclear RNA-binding protein
ADATs: adenosine deaminases that act on tRNAs
TadA: tRNA adenosine deaminase
AID: activation-induced cytidine deaminase
APOBEC: apolipoprotein B mRNA editing enzyme
AADs: AID/APOBEC family of deaminases
SNADs: secreted deaminases
CDAT8: cytidine deaminase acting on tRNA base C8
WT1: Wilm’s tumor 1 human transcript
hnRNP K: heterogeneous nuclear ribonucleoprotein K
ZDD: zinc-dependent cytidine (C) or deoxycytidine (dC) deaminase domain
A1CF: RNA binding protein cofactor
dsRBD: dsRNA binding domain
R domain: single-stranded RNA binding domain
SINE: short interspersed element
LINE: long interspersed nuclear element
GIREMI: Genome-independent Identification of RNA Editing by Mutual Information
lncRNAs: long noncoding RNAs
miRNAs: microRNAs
NARF: nuclear prelamin A recognition factor
SHM: somatic hypermutation
CSR: class switch recombination
Vif: virion infectivity factor
SIV: Simian immunodeficiency virus
AAV-2: adeno-associated virus type 2
MLV: murine leukemia virus
EIAV: equine infectious anemia virus
FV: foamy virus
RVFV: Rift Valley fever virus
LCMV: lymphocytic choriomeningitis virus
HCV: hepatitis C virus
ALS: amyotrophic lateral sclerosis
OPTN: optineurin
RAP2A: RAS related protein 2a (a member of the RAS oncogene family)
AMFR: autocrine motility factor receptor
CPEB1: cytoplasmic polyadenylation element binding protein 1
TCGA: The Cancer Genome Atlas
MDM2: transformed mouse 3T3 cell double minute 2/MDM2 proto-oncogene
PKCζ: protein kinase Cζ
AZIN1: antizyme inhibitor 1
ER: estrogen receptor
MMP2: matrix metalloproteinase 2
FGFR3: fibroblast growth factor receptor 3
CPTAC: Clinical Proteomic Tumor Analysis Consortium
